# Cesarean scar dehiscence in early puerperium and influence of barbed suture: tridimensional ultrasound evaluation in a randomized clinical study

**DOI:** 10.1590/acb399124

**Published:** 2024-11-29

**Authors:** Newton de Paula Ishikawa, Gabriela Ewerling Souza, Thays Andressa Albuquerque Monteiro, Albert Schiaveto de Souza, Ricardo Dutra Aydos, Durval Batista Palhares

**Affiliations:** 1 Universidade Federal de Mato Grosso do Sul – Graduate Program in Health and Development in Brazil’s Center-West Region – Campo Grande (MS) – Brazil.; 2 Universidade Federal de Mato Grosso do Sul – Medical Residency Program in Gynecology and Obstetrics – Hospital Universitário Maria Aparecida Pedrossian – Campo Grande (MS) – Brazil.; 3 Universidade Federal de Mato Grosso do Sul – School of Medicine– Campo Grande (MS) – Brazil.

**Keywords:** Cesarean Section, Diagnostic Techniques, Obstetrical and Gynecological, Suture Techniques

## Abstract

**Purpose:**

This study investigated the hypothesis of early dehiscence of hysterorrhaphy as the initial stage of post-cesarean uterine scar defects, examining the possible influence of barbed suture in this process.

**Methods:**

This longitudinal, prospective, double-blind study included 54 pregnant women with no history of cesarean section, randomized into two suture groups: #0 polyglactin or #1 barbed PDS threads. Sutures were continuous, unlocked, involved the entire myometrium in a single layer, and included the endometrium. Tridimensional transvaginal ultrasonography was performed on the second day postpartum to investigate scar dehiscence and measure its maximal width.

**Results:**

The groups had 29 and 25 participants, respectively. Ages: 18–37 (mean 25.80 ± standard error of the mean 0.69) years old. Groups were homogeneous for labor duration, cervical thickness, edge-to-os distance, retroversion, amniotic sac rupture, and additional hemostatic sutures required. Uterine retroversion accounted for 7.4% of cases. Dehiscence was observed in 68.5% (3.98 ± 0.57 mm). The only factor correlating (positively) with dehiscence width was myometrial thickness, whether proximal or distal.

**Conclusions:**

Suture type had no influence on early dehiscence, which occurred at the same rate as published niche formation rates. Tridimensional ultrasound proved effective for evaluating dehiscence.

## Introduction

Owing to decreasing surgical risks, improved hospital resources, and excellent esthetic results, cesarean section has become the most frequently performed surgery worldwide. Although the World Health Organization recommends that cesarean section rates do not exceed 12–15%, a 2021 survey reported that 21% of deliveries employed this method, with rates projected to rise to 29% by 2030^1^.

Obstetric implications of the method in subsequent pregnancies have long been recognized, among them low placental insertion, gestation in a cesarean scar, uterine rupture, and placental accretion. Only in the past decade, however, long-term gynecological complaints have drawn closer attention, including intermenstrual uterine bleeding, infertility, dyspareunia, and chronic pelvic pain. All these complaints share a common underlying feature: niche, or isthmocele—a type of defect in the cesarean scar region. With rates of 60% after the first cesarean delivery, 80% after the second, and virtually 100% after the third one, niche constitutes the most frequent complication of the world’s most performed surgery^2^.

Niche is defined as an indentation with a depth of at least 2 mm in the myometrium at the site of a cesarean scar^3^. The most commonly available imaging methods for its diagnosis are transvaginal ultrasound and hysterosonography^4^, especially with the use of tridimensional (3D) technology, which increases sensitivity, yielding better visualization of niche dimensions^5^.

While a number of risk factors have been identified, knowledge of niche pathophysiology remains limited. Rapid, pronounced uterine involution after delivery may negatively impact suture quality, potentially leading to suture loosening and early dehiscence^6^^,^ 7, as often observed in re-operative interventions in the early postpartum period, with consequent niche development.

The use of barbed suture in cesarean sections is associated with lower blood loss and shorter surgical time^8^, but its influence on niche formation remains unclear. Barbed sutures are synthetic, absorbable monofilaments of polydioxanone containing triclosan, a bactericide.

This investigation compared barbed polydioxanone (PDS) suture against #0 braided polyglactin suture, the thread most often used in obstetric and gynecological procedures, and widely available in maternity wards for uterine suturing in cesarean sections.

The presence of barbs reduces tensile strength, relative to barb-free material of the same gauge. The tensile strength of #0 polyglactin suture is equivalent to that of #1 barbed PDS thread.

The purposes of this study were to employ 3D ultrasonography to seek evidence of early dehiscence of post-cesarean uterine scar and evaluate the influence of barbed suture on its occurrence—a potential factor in the pathophysiology of niche formation.

## Methods

### Ethical issues

This investigation complied with Resolution no. 466/12 issued by the National Health Council of the Brazilian Ministry of Health and commenced after approval by the Ethics Committee of the Universidade Federal de Mato Grosso do Sul (protocol 5,769,468), the HUMAP-EBSERH Teaching and Research Management Office (opinion 82, issued September 21, 2022), and inclusion in the Plataforma Brasil registry.

Subjects agreed to participate by providing written informed consent.

### Procedures

From February to September 2023, a longitudinal study of 54 pregnant women scheduled for cesarean section at the HUMAP-EBSERH obstetric emergency service was conducted. Participants were randomized for a double-blind study designed to compare two surgical sutures, with 29 participants in the polyglactin 0 group and 25 in the barbed PDS group. Incidence and maximal width of uterine suture dehiscence were evaluated using 3D transvaginal ultrasound two days after the first cesarean section.

### Primary and secondary outcomes

The primary outcome was defined as the overall incidence of scar dehiscence on the second day postpartum. The difference in dehiscence incidence and maximal dehiscence width between suture types was a secondary outcome measure.

Surgical sutures characteristics are described in Table 1.

**Table 1:** Suture characteristics.

	Polyglactin	Barbed PDS
Material	Synthetic	Synthetic
Composition	Lactic acid: 70% Glycolic acid: 30% Polyglactin-coated	Polydioxanone (PDS)
Structure	Braided	Monofilament
Shape	Round-bodied	Barbed
Gauge	0	1
Degradation	Absorbable	Absorbable
Absorption mode	Hydrolysis	Hydrolysis
Tensile strength	28–35 days	75% after 14 days 55% after 42 days
Absorption time	56–70 days	120–180 days
Needle	½ circle, 36.4 mm long	CT-1 Plus, ½ circle, 36.4-mm long

Source: Adapted from Barros et al.^9^.

### Criteria for inclusion

18 years old or older.

Pregnant women with no history of cesarean sections.

### Criteria for exclusion

Uncontrolled gestational diabetes, peripartum anemia, chronic use of corticosteroids, multiple pregnancies, polyhydramnios, residing outside Campo Grande county (MS), Brazil, Müllerian anomalies, placenta previa, chronic inflammatory disease, chorioamnionitis, fever of any nature, infection in the incision topography, myoma at the hysterotomy site, lower-segment transverse section; more than two additional hemostatic sutures, endometritis, uterine re-suturing in the postpartum period, corticosteroid use up to 30 days postpartum.

### Prohibited medications during the study

Corticosteroids in the first month after the procedure.

### Subjects were not excluded if

- Using prophylactic antibiotics;
- Amniorrhexis showed no evidence of chorioamnionitis.

### Participants were excluded from the study after cesarean section if

- Undergoing a modification of the standardized surgical technique;
- Receiving more than two additional hemostatic sutures during uterine closure;
- Revoking the written consent.

### Intervention

The cesarean sections were performed by a team of certified obstetricians from the HUMAP-EBSERH maternity sector who were fully aware of the mandatory times involved in the procedure steps—namely, uterine opening using the Kerr maneuver; not exteriorizing the uterus for hysterorrhaphy; randomization of sutures; continuous, unlocked, single-layer suturing with endometrial inclusion; visualization of ovaries, hemostasis review; not closing visceral or parietal peritoneum; administration of prophylactic antibiotics for 24 h; and administration of oxytocin for a maximum of 24 h. Decisions on the remaining surgical times, considered as not affecting uterine healing, were left to the surgeon’s discretion. Any modification to the technique was reported to the lead investigator.

### Ultrasonographic evaluation

Examinations were performed on the second day postpartum. The transvaginal route was chosen for being of shorter duration, less uncomfortable, providing better quality, and not involving the surgical scar of the abdominal wall, thus avoiding ultrasound artifacts in this region.

### Device

All ultrasound exams employed the same HM70 EVO device (Samsung Medison, Republic of Korea), with a volumetric transvaginal IPX7 probe (8 MHz).

### Early postpartum ultrasonographic evaluation

With the patient’s bladder empty or holding a small amount of urine, an 8-MHz volumetric transvaginal probe was employed, covered with a condom containing gel only inside. Bidimensional ultrasound examination was video-recorded as a single left-to-right sweep on the patient, followed by a 90° rotation to the left to acquire posterior-to-anterior views of the uterus.

For 3D ultrasound examination, the 3D volume box was parameterized with a scan angle of 120° and maximum-quality scanning speed. The box was dimensioned to encompass the entire hysterorrhaphy.

Evaluation of the 3D block began in multiplanar mode, employing the RealisticView feature and left-to-right illumination at an incidence angle of roughly 45°. The best section was selected by identifying the hysterorrhaphy and applying the MultiSlice feature, then adjusting zoom so that the hysterorrhaphy filled most of window A without hiding the scar edges. Once the best section was selected, the box was adjusted to be tangent to the central point shared by the three windows.

In the presence of dehiscence, the largest measurement was taken in 3D mode using window C.

### Randomization

Randomization into blocks of 10 at a 1:1 ratio was carried out in advance by an individual blinded to the investigation. Prior to each hysterorrhaphy, suture type was selected by random draw by the surgical team. The results were individually placed in sealed envelopes, together with medical record number and intervention date.

### Blinding

Suture type was known to the surgical team and the nursing technician who opened the suture, but not to the patient, ultrasound examiner, or the nursing team in care. The envelope holding this information was not opened before the time of statistical analysis.

### Analysis of the results

Student’s t-test was performed to compare patients with presence vs. absence of dehiscence regarding quantitative variables. The test was also used to compare subjects in labor vs. not in labor, patients with intact vs. ruptured membranes, and subjects with vs. without uterine retroversion, for the maximal dehiscence widths observed. Student’s t-test was also employed to compare patients receiving Stratafix *vs.* polyglactin suture based on quantitative data. The χ^2^ test was performed to evaluate associations between categorical variables and dehiscence and between categorical variables and suture type. Linear correlation between quantitative variables and maximal dehiscence width was evaluated using Pearson’s linear correlation test. Other data underwent descriptive statistical analysis or were presented as tables and graphs. Statistical analysis was performed using Statistical Package for the Social Sciences software, v. 24.0, adopting a significance level of 5%^10^.

## Results

Participants’ characteristics are shown in Table 2, age ranged from 18 to 37 (25.80 mean ± 0.69 standard error of the mean) years old. Most patients were not in labor when cesarean section was performed (70.4%; n = 38). Mean duration of labor was 2.81 ± 0.64 h; mean dilation was 1.87 ± 0.33 cm; and mean cervical thickness was 31.69 ± 1.67 mm. Most patients had intact membranes at the time of delivery (72.2%; n = 39). Mean proximal and distal myometrial thickness measurements were 6.76 ± 0.72 mm and 5.44 ± 0.45 mm, respectively. Mean edge-to-os distance was 46.35 ± 2.52 mm, and mean number of additional hemostatic sutures required was 0.17 ± 0.07. Uterine retroversion was observed in only 7.4% of patients (n = 4). Most subjects presented early uterine scar dehiscence on the second day postpartum (68.5%; n = 37). Mean measurement of maximal dehiscence width was 3.98 ± 0.57 mm. Polyglactin suture was employed in 53.7% of patients (n = 29), while Stratafix was used in 46.3% (n = 25).

**Table 2:** Sample characteristics.

Variable	Mean ± SEM or % (n)
**Age (18–37 years old)**	25.80 ± 0.69
**In labor**	
No	70.4 (38)
Yes	29.6 (16)
**Duration of labor (h)**	2.81 ± 0.64
**Dilation (cm)**	1.87 ± 0.33
**Effacement (mm)**	31.69 ± 1.67
**Membranes**	
Intact	72.2 (39)
Ruptured	27.8 (15)
**Proximal myometrial thickness (mm)**	6.76 ± 0.72
**Distal myometrial thickness (mm)**	5.44 ± 0.45
**Edge-to-os distance (mm)**	46.35 ± 2.52
**Additional hemostatic sutures**	0.17 ± 0.07
**Retroverted uterus**	
No	92.6 (50)
Yes	7.4 (4)
**Dehiscence**	
No	31.5 (17)
Yes	68.5 (37)
**Maximal dehiscence width (mm)**	3.98 ± 0.57
**Suture thread**	
Stratafix	46.3 (25)
Polyglactin	53.7 (29)

SEM: standard error of the mean. Source: Elaborated by the authors.

As shown in Table 3, no significant relationships were found between the variables investigated and presence or absence of uterine dehiscence postpartum (Student’s t-test or χ^2^ test, 0.136 < *p* < 0.909).

**Table 3:** Relationships between variables and uterine dehiscence after cesarean delivery*.

Variable	Dehiscence		*p*-value
	No	Yes	
**Age (years old)**	26.35 ± 1.37	25.54 ± 0.80	0.592
**In labor**			
No	36.8 (14)	63.2 (24)	0.191
Yes	18.8 (3)	81.3 (13)	
**Duration of labor (h)**	1.59 ± 0.38	3.38 ± 0.83	0.149
**Dilation (cm)**	1.59 ± 0.38	2.00 ± 0.45	0.489
**Effacement (mm)**	32.35 ± 2.75	31.38 ± 2.10	0.789
**Membranes**			
Intact	25.6 (10)	74.4 (29)	0.136
Ruptured	46.7 (7)	53.3 (8)	
**Proximal myometrial thickness (mm)**	6.88 ± 0.64	6.70 ± 1.01	0.909
**Distal myometrial thickness (mm)**	4.82 ± 0.38	5.73 ± 0.63	0.356
**Edge-to-os distance (mm)**	42.29 ± 4.17	48.22 ± 3.14	0.280
**Additional hemostatic sutures**	0.06 ± 0.06	0.22 ± 0.10	0.186
**Retroverted uterus**			
No	32.0 (16)	68.0 (34)	0.772
Yes	25.0 (1)	75.0 (3)	

* Values are expressed as mean ± standard errors of the mean for quantitative variables or relative frequencies (followed by absolute frequencies) for categorical variables;

*p*: Student’s t-test (quantitative variables) or χ^2^ test (categorical variables). Source: Elaborated by the authors.

**Figure 1:**
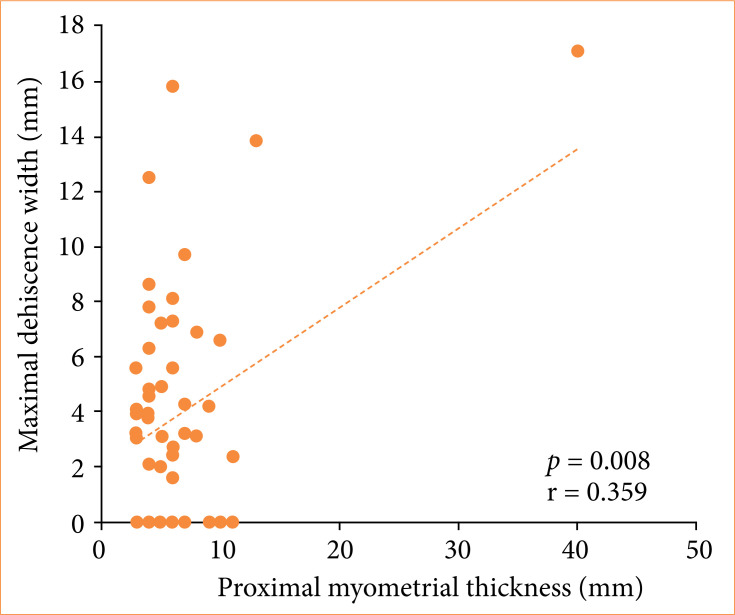
Relationships between maximal dehiscence width and proximal myometrial thickness.

Source: Elaborated by the authors.

**Figure 2:**
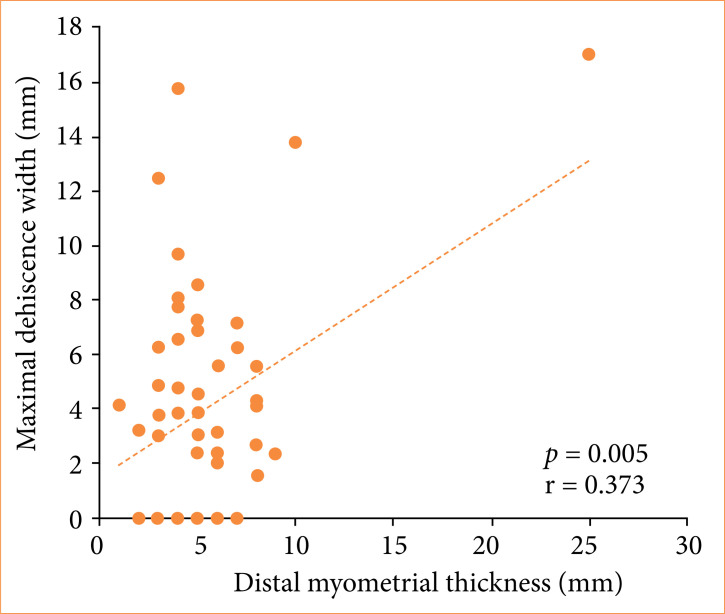
Relationships between maximal dehiscence width and distal myometrial thickness.

Source: Elaborated by the authors.

The relationships between variables and maximal dehiscence width are shown in Table 4. Only myometrial thickness correlated linearly, albeit weakly to moderately, with maximal dehiscence width measurement (Pearson’s linear correlation test; proximal myometrial thickness: *p* = 0.008, r = 0.359; distal myometrial thickness: *p* = 0.005; r = 0.373; Figs. 1 and 2, respectively). No significant relationships were found between maximal dehiscence width and other variables (Pearson’s linear correlation test or Student’s t-test, 0.438 < *p* < 0.919).

**Table 4:** Relationships between variables and maximal dehiscence width after cesarean delivery*.

Variable	Maximum dehiscence (mm)
**Age (years old)**	p = 0.907; r = 0.016
**In labor**	
No	3.77 ± 0.74
Yes	4.48 ± 0.78
*p*	0.575
**Duration of labor (h)**	*p* = 0.438; r = 0.108
**Dilation (cm)**	*p* = 0.771; r = 0.041
**Effacement (mm)**	*p* = 0.919; r = 0.014
**Membranes**	
Intact	4.35 ± 0.70
Ruptured	3.02 ± 0.90
*p*	0.297
**Proximal myometrial thickness (mm)**	*p* = 0.008; r = 0.359
**Distal myometrial thickness (mm)**	*p* = 0.005; r = 0.373
**Edge-to-os distance (mm)**	*p* = 0.468; r = 0.101
**Additional hemostatic sutures**	*p* = 0.605; r = 0.073
**Retroverted uterus**	
No	3.92 ± 0.60
Yes	4.78 ± 1.80
*p*	0.695

* Results are expressed as *p*- and r-values (Pearson’s linear correlation test) or as mean ± standard errors of the mean;

p: Student’s t-test (in comparative tests); r: linear correlation coefficient. Source: Elaborated by the authors.

No significant differences were detected between suture type and the variables investigated (Student’s t-test or χ^2^ test, 0.053 < *p* < 0.920; Table 5).

**Table 5:** Relationships between variables and suture type*.

Variable	Suture		*p*-value
	Stratafix	Polyglactin	
**Age (years old)**	25.72 ± 0.88	25.86 ± 1.06	0.920
**In labor**			
No	80.0 (20)	62.1 (18)	0.150
Yes	20.0 (5)	37.9 (11)	
**Duration of labor (h)**	1.52 ± 0.69	3.93 ± 1.00	0.053
**Dilation (cm)**	1.52 ± 0.50	2.17 ± 0.44	0.328
**Effacement (mm)**	32.44 ± 2.50	31.03 ± 2.27	0.678
**Membranes**			
Intact	76.0 (19)	69.0 (20)	0.565
Ruptured	24.0 (6)	31.0 (9)	
**Proximal myometrial thickness (mm)**	6.40 ± 0.59	7.07 ± 1.24	0.646
**Distal myometrial thickness (mm)**	5.12 ± 0.44	5.72 ± 0.75	0.510
**Edge-to-os distance (mm)**	47.12 ± 3.50	45.69 ± 3.66	0.781
**Additional hemostatic sutures**	0.08 ± 0.08	0.25 ± 0.11	0.219
**Retroverted uterus**			
No	88.0 (22)	96.6 (28)	0.232
Yes	12.0 (3)	3.4 (1)	

* Values are expressed as mean ± standard errors of the mean for quantitative variables or relative frequencies (followed by absolute frequencies) for categorical variables;

*p*: Student’s t-test (quantitative variables) or χ2 test (categorical variables). Source: Elaborated by the authors.

Suture type had no significant influence on the presence or absence of dehiscence (χ^2^ test, *p* = 0.939) or on maximal dehiscence width (Student’s t-test, *p* = 0.289) (Table 6).

**Table 6:** Relationships between suture type and dehiscence and between suture type and largest dehiscence width*.

Variable	Suture		*p*-value
	Stratafix	Polyglactin	
**Dehiscence**			
No	32.0 (8)	31.0 (9)	0.939
Yes	68.0 (17)	69.0 (20)	
**Maximal dehiscence width (mm)**	3.33 ± 0.68	4.54 ± 0.87	0.289

* Values are expressed as relative frequencies (followed by absolute frequencies) for dehiscence or mean ± standard errors of the mean for maximal dehiscence width;

p: χ2 test (dehiscence) or Student’s t-test (maximal dehiscence width). Source: Elaborated by the authors.

## Discussion

Countless maternal and fetal lives have been saved by cesarean section, but a 2021 study revealed that the 21% rate reported had reached nearly double the figure recommended by the World Health Organization (10–15%). The procedure is even more frequent in East Asia (63%), Latin America–Caribbean (54%), West Asia (50%), North Africa (48%), South Europe (47%), and Australia–New Zealand (45%)^1^.

**Figure 3:**
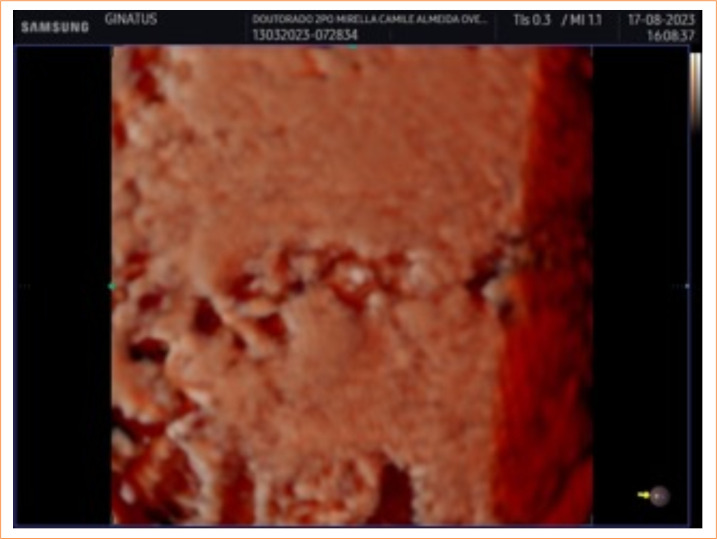
Dehiscence.

Source: Elaborated by the authors.

Commensurate with the increasing rates of cesarean section, the incidence of uterine scar defects (niche) has also grown, at widely varying rates, depending on the definition adopted, the diagnostic method used, and on clinical characteristics such as number of cesareans. A systematic review has shown that these rates range from 20 to 90%, regardless of the imaging technique employed4. The hysterorrhaphy dehiscence rate of 68.5% (n = 37) found in the present study is consistent with the hypothesis, advanced by Poidevin in 1961^11^, that uterine wounds will not have closed within the first few post-intervention days (Fig. 3). Although the pathophysiology of dehiscence is not entirely clear, the present demonstration of its early occurrence post-hysterorrhaphy, previously hypothetical, may prove relevant for a fuller understanding of niche formation. Given that most participants (70.4%) in the present study were not in labor or were in its early stages (mean 2.81 ± 0.64 h), the general dehiscence rate might be even higher than 68.5%, since the risk of uterine scar defects increases when a cesarean section is performed before labor has initiated or when in its early stages, with limited cervical dilation^12^^,^ 13.

In addition to revealing high dehiscence rates, the present results demonstrated a positive linear correlation between proximal and distal myometrial thickness and dehiscence width. This finding lends support to the view that performing a cesarean section before the onset of labor can influence uterine wound healing, since the absence of cervical dilation may hinder drainage of the uterine cavity, requiring more vigorous uterine activity and leading to myometrial thickening, with consequent thinning at the incision site^14^.

The postpartum uterine healing process follows a unique dynamic, in that no other organ undergoes such rapid, extreme physiological changes. Under such singular conditions, wound healing failure does not constitute a rare event. A study of uterine involution in the early postpartum with ultrasound exams on the first, second, and seventh days found reductions in uterine volume, longitudinal diameter, anteroposterior diameter, and transverse diameter by 44.8, 20.9, 11.8, and 20%, respectively. As reported by Edwards and Ellwood^15^, the difference between uterine involution after cesarean section and after normal delivery proved significant on the second day, with more pronounced involution in mothers subjected to cesarean section—a reason for selecting this timepoint in the present investigation.

In a systematic review, Verberkt et al.^13^ grouped risk factors as follows: patient-related (*e.g.*, number of cesareans, body mass index, uterine retroversion), labor-related (prolonged labor, exceeding 5 h; degree of dilation; incision height; lower-segment thickness), and surgery-related.

The influence of surgical approaches has gained increased attention with the growing body of evidence of their effect on niche formation—highly relevant knowledge for the prevention of this defect.

To date, no consensus has been reached on the best surgical approach for performing cesarean sections, drawing on the results of randomized clinical trials or the combination of possible intervening factors^16^. In addition to the suture type selected, uterine closure techniques can incorporate variations, such as single- *vs.* double-layer closure and anchored vs. unloking suture, comprising full or partial thickness, and including the endometrial layer or otherwise. The decision on choice of technique is informed by a few clinical conditions, such as degree of cervical dilation, labor duration, number of cesareans, uterine position, and integrity of the amniotic sac.

In recent decades, two randomized clinical studies—the Coronis^17^ and the Caesar trials^18^ —failed to detect significant differences between the interventions investigated. In the United Kingdom, the National Institute for Health and Care Excellence allows surgeons to choose between single- or double-layer hysterorrhaphy. In other countries, including the Netherlands and Belgium, an overwhelming majority of surgeons (92.2%) employ continuous single-layer closure (96.2%) without any anchoring (87.1%)^19^. Ultimately, absence of consensus has allowed surgeons to adopt their preferred cesarean section and uterine closure techniques^16^.

In a review study, Tulandi and Cohen^20^ reported that both double-layer closure and endometrial inclusion have been associated with lower prevalence of cesarean scar defects. However, these findings differed from the observations made by Roberge et al.^21^ and Vikhareva Osser and Valentin^22^ that single-layer closure was not associated with the development of pronounced scar defects. Impaired blood perfusion and relative hypoxia of the scar, stemming from the mechanical tension of hysterorrhaphy, may explain the increased incidence of scar in anchored sutures^14^^,^ 23.

Polyglactin suture, adopted for the unlocked, continuous, endometrium-inclusive single-layer uterine closure performed in the present study, is the most frequently used surgical thread during cesarean delivery^24^. Furthermore, the material is suitable for all lower-segment thicknesses—an advantageous feature in the current sample, since the cesarean sections were performed at an emergency ward.

The choice of barbed suture for comparative purposes drew on studies reporting that this material required shorter suturing times, without compromising hemostasis^24^, and was also based on the observation, by Alessandri et al.^25^, of a lower (albeit not significantly) incidence of early dehiscence for barbed *vs.* polyglactin suture. (The investigators remarked that a greater sample size might be required in order to reach significance.) This disparity in results might stem from their use of two-layer suturing, whereas single-layer suturing was employed in the present investigation.

Despite their reported advantages, barbed sutures are more costly than other surgical thread options, representing a barrier to their adoption in resource-limited regions.

The diagnostic method also influences the observation of scar defects. In the present study, selection of the second postoperative day took into account the fact that mothers would still be recovering from delivery and eliminating lochia. For many reasons, it was not possible to perform saline hysterosonography at this stage, despite the technique’s effectiveness in the observation of cesarean scars. 3D endovaginal ultrasound was employed instead, for providing rapid, accurate, comfortable examination. Because the irregular shape of dehiscence would make measurements more complex, only width measurements were taken.

Group compositions were homogeneous regarding factors, such as retroversion, age, labor duration, and incision height, that might potentially have confounded comparisons. Incision height can affect the outcome, because presence of mucus in the scar can induce dehiscence of myometrial edges^26^, especially in low incisions reaching the cervical region, more frequently performed when dilation is greater than 3 cm^22^.

Non-closure of the deep myometrial layer (also reported as a potential predisposing factor for niche development) was not considered as a variable in the present investigation, which standardized suturing to encompass the entire myometrial thickness, including the endometrium.

Could early hysterorrhaphy dehiscence have histopathological repercussions? There is scant literature describing niche histopathological features^6^. Niche is typically described as containing mucus, with the endocervical mucosa often presenting a cystically dilated and/or atrophic aspect, or with a disorganized endometrial mucosa in the lower uterine segment^27^. Histological examinations have revealed fibrotic tissue in 78.9% of women who underwent cesarean section and significantly lower residual myometrial muscle density than in healthy myometrium in these patients^28^. Although these characteristics are common to any scar tissue, their occurrence does not rule out adoption of secondary-intention healing^28^.

In 120 consecutive symptomatic patients treated with hysteroscopy, fibrosis and scar tissue were found in 90% of cases and chronic inflammatory infiltration of the endocervix in 10%. Regenerative epithelial atypia and fibroblastic stromal reaction are frequent, exclusive features of scars containing niches. No granulomatous reaction, significant inflammation, or bleeding have been observed^27^.

Congested endometrium above the scar recess (61%) has been also reported, as have moderate-to-severe lymphocytic infiltration (65%), capillary dilation (65%), free red blood cells in the scar endometrial stroma (59%), iatrogenic adenomyosis confined to the scar (28%), and polyps^29^. In women with symptomatic niches, the presence of CD138 protein on immunohistochemical analysis indicates chronic, rather than acute, inflammation^27^.

A question raised elsewhere is whether the polyps reported in 23.7% of cases investigated were polyps proper or solely an overlying layer of disorganized mucosa. Another question is whether adenomyosis, reported in 11.8% of patients, is authentic or iatrogenic (resulting from entrapment of endometrial glands and stroma in the scar, while cystic endocervical inclusions proved infrequent). These features appear to be related to the presence of niche and not to the cesarean scar itself, since they were not observed in the absence of niche^27^.

### Strengths of this investigation

This novel, randomized, double-blind study (for patient and evaluator) looked for evidence of uterine scar dehiscence on the second day postpartum. Patients were subsequently on follow-up to investigate whether dehiscence would evolve to niche by the end of the postpartum period.

### Limitations of the investigation

Low number of patients. A larger sample might reveal significant differences between groups regarding dehiscence rates and dimensions.

Participants continue to receive ultrasound follow-up to investigate whether early postpartum dehiscence has evolved to niche at the end of puerperium (45–55 days postpartum). Dehiscence at such early stages requires further investigation. Prevention of niche formation may constitute the first step toward reducing the currently alarming rates of niche and its consequences.

## Conclusion

Early post-cesarean dehiscence of uterine sutures was found to occur at the same rate as niche formation reported in the literature—two tissue alterations that may be related. 3D endovaginal ultrasound proved effective for evaluating early dehiscence. Use of barbed suture had no influence on the high rates of this dehiscence or its dimensions. The only factor correlating (positively) with dehiscence width was myometrial thickness. No postoperative complications were reported. Subsequent follow-up is expected to yield further data on possible evolution of early dehiscence.

## Data Availability

Data will be available upon request.
